# Implant-based dental rehabilitation in head and neck cancer patients after maxillofacial reconstruction with a free vascularized fibula flap: the effect on health-related quality of life

**DOI:** 10.1007/s00520-022-06944-4

**Published:** 2022-03-17

**Authors:** Johannes N. Lodders, Gustaaf J. C. van Baar, Marije R. Vergeer, Femke Jansen, Engelbert A. J. M. Schulten, Birgit I. Lissenberg-Witte, Irma M. Verdonck-de Leeuw, Tymour Forouzanfar, Frank K. J. Leusink

**Affiliations:** 1 Amsterdam UMC and Academic Centre for Dentistry Amsterdam (ACTA), Vrije Universiteit Amsterdam, Department of Oral and Maxillofacial Surgery/Oral Pathology, Cancer Center Amsterdam, De Boelelaan 1117, 1081 HV Amsterdam, The Netherlands; 2 Department of Radiation Oncology, Amsterdam UMC, VU University Medical Center, De Boelelaan 1117, 1081 HV Amsterdam, The Netherlands; 3 Department of Otolaryngology-Head and Neck Surgery, Amsterdam UMC, Vrije Universiteit Amsterdam, Cancer Center Amsterdam, De Boelelaan 1117, 1081 HV Amsterdam, The Netherlands; 4 Department of Epidemiology and Data Science, Amsterdam UMC, Vrije Universiteit Amsterdam, PO Box 7057, 1007 MB Amsterdam, The Netherlands; 5 Department of Behavioural and Movement Sciences, Section Clinical Psychology, Vrije Universiteit Amsterdam Public Health, Van der Boechorststraat 7, 1081 BT Amsterdam, The Netherlands

**Keywords:** Quality of life, Rehabilitation, Dental implants, Fibula free flap, Head and neck cancer

## Abstract

**Purpose:**

To evaluate the effect of implant-based dental rehabilitation (IDR) on health-related quality of life (HRQoL) in head and neck cancer (HNC) patients after reconstruction with a free vascularized fibula flap (FFF).

**Methods:**

Eligible patients were identified by retrospectively reviewing the medical records of patients treated in Amsterdam UMC-VUmc. HRQoL data were used from OncoQuest, a hospital-based system to collect patient-reported outcome measures in routine care. Data were used of the EORTC QLQ-C30 and QLQ-H&N 35 before FFF reconstruction (T_0_) and after completing IDR (T_1_). Data were statistically analysed with the chi-square test, independent samples *t* test and linear mixed models.

**Results:**

Out of 96 patients with maxillofacial FFF reconstruction between January 2006 and October 2017, 57 patients (19 with and 38 without IDR) had HRQoL data at T_0_ and T_1_. In the cross-sectional analysis, patients with IDR scored significantly better at T_0_ and T_1_ on several EORTC domains compared to the patients without IDR. Weight loss was significantly different in the within-subject analysis between T_0_ and T_1_ for patients with IDR (*p* = 0.011). However, there were no significant differences in the mean changes of all the EORTC QLQ-C30 and EORTC QLQ-H&N35 scores between the defined timepoints for patients with IDR compared to those without.

**Conclusions:**

In this study, no differences were found in the course of HRQoL in HNC patients who had undergone IDR after maxillofacial FFF reconstruction, compared to those who had not. Patients should be preoperatively informed to have realistic expectations regarding the outcome of IDR.

## Introduction

Surgical treatment of oral cavity tumours may lead to complex segmental mandibular or maxillary defects [1, 2] resulting in functional impairment with regard to mastication, speech and swallowing. The vascularised fibula free flap (FFF) has become the standard of care for reconstruction of mandibular defects [1, 3] and is also the preferred flap for reconstruction of maxillary defects [4].

Maxillofacial reconstruction with an FFF after ablative oncological surgery optimises function and aesthetics, with acceptable results regarding flap survival, donor site morbidity and perioperative complications [5, 6]. However, patients who have undergone FFF reconstruction expect restoration of oral function close to their pre-surgical state [7]. To fulfil this wish, implant-based dental rehabilitation (IDR) can contribute to improve functional and aesthetic outcomes [7, 8] and may become a standard part of the total rehabilitation plan [9].

In the literature, it is shown that, although a minority of head and neck cancer (HNC) patients commence IDR after FFF reconstruction [10, 11], good results can be achieved regarding dental implant survival, dental implant success and percentage of functional prosthetic rehabilitations [9–11]. However, evidence on the effect of IDR on health-related quality of life (HRQoL) in this patient group is limited [9]. Four studies showed minor improvements in HRQoL using validated questionnaires in patients who underwent IDR after FFF reconstruction [12–15]. One prospective trial reported a clear benefit of IDR on HRQoL with validated questionnaires [16]. Limitations of these studies were the lack of a control group^13^,[16] and that HRQoL was measured at one timepoint [12, 14]. In addition, most studies on HRQoL included benign pathology [12–14, 16].

Therefore, the purpose of this study was to evaluate the effect of IDR on HRQoL in HNC patients after FFF reconstruction, measured at different timepoints and using a control group.

## Materials and methods

### Study design and study population

In this retrospective cohort study, two databases were searched to identify patients eligible for the study: a clinical database of the Department of Oral and Maxillofacial Surgery/Oral Pathology, Amsterdam UMC-VU Medical Center (VUmc) in Amsterdam, The Netherlands, and a database with patient-reported outcome measures (OncoQuest) of the Department of Otolaryngology—Head and Neck Surgery and the Department of Radiation Oncology of Amsterdam UMC—VUmc, Amsterdam, The Netherlands. OncoQuest comprises patient-reported outcome measures (PROMs) that are gathered as part of routine patient care before the start of oncological treatment and during follow-up visits via a touch screen computer [17].

Patients were included in this study if they were (1) diagnosed with HNC; (2) had undergone maxillofacial reconstruction with an FFF between January 2006 to October 2017; and (3) aged 18 years or older; and (4) if data regarding HRQOL was available, of which (5) the patient provided informed consent to use these data for research purposes. Patients with benign diseases and free flaps other than FFF were excluded from this study.

All included patients were allocated in two groups: (a) FFF reconstruction without implant-based dental rehabilitation (without IDR) and (b) FFF reconstruction followed by implant-based dental rehabilitation (IDR).

To qualify for IDR, patients needed to have unsatisfactory oral function and/or aesthetics that could be improved with dental rehabilitation, and to have been free of disease or recurrence for at least 12 months after completion of all adjuvant therapy.

### Demographic and clinical variables

Demographic and clinical characteristics such as age, gender, tobacco and alcohol use, ASA classification, radiotherapy data (with or without concurrent chemotherapy), dental status, number of FFF segmentations, type of mandibular defect [1] and type of maxillary defect [2] were collected from the medical information system. Disease stage and tumour entity were gathered as histopathological data. The amount of dental implants and information regarding the dental superstructures were assessed.

### HRQoL measurements

HRQoL was evaluated using the European Organization for Research and Treatment of Cancer Quality of Life Core 30 (EORTC QLQ-C30)[18] and the module specifically designed for HNC patients (EORTC QLQ-H&N 35) [19].

The EORTC QLQ-C30 contains one global QoL scale, five functional scales, three symptom scales and six single items. The EORTC QLQ-H&N 35 module contains seven symptom scales and 11 single items. A higher score for global QoL scale and functional scales reflects a better level of functioning. A higher score for symptom scales reflects a higher level of symptoms. All scales and single items are converted to a score from 0 to 100.

### Statistical analysis

The SPSS Software package (version 20.0 IBM, Armonk, NY, USA) was used for statistical analysis.

To identify differences in demographic parameters between the defined groups, the independent *t* test and chi-square test were used. If the expected counts were less than five, Fisher’s exact test was used.

Two timepoints were defined: T_0_: HRQoL data for the period from 6 months before FFF reconstruction until the FFF reconstruction, and T_1_: HRQoL data in the period after completing IDR (i.e. after placement of the dental superstructure). For patients who did not undergo IDR, T_1_ was defined as the period after FFF reconstruction. If HRQoL data was available for multiple timepoints after T_1_, HRQoL data closest to 2 years after completing oncological treatment was used. This specific timepoint was used as T_1_ because global QoL seems to gradually improve until 1 year after finishing oncological treatment in HNC patients [20].

For cross-sectional analysis at T_0_, the chi-square test was used for dichotomous variables and independent samples *t* test for continuous variables.

Longitudinal linear mixed (LMM) models were used for within-subject analysis to analyse the course of HRQoL in patients who had undergone IDR after FFF reconstruction versus those who did not, as well as to analyse differences in the course of HRQoL between these patients. The within-subject model included a fixed effect for time and a random effect for subject, and the between-subject model additionally included a fixed effect for group and the interaction between time and group.

## Results

**Fig. 1:**
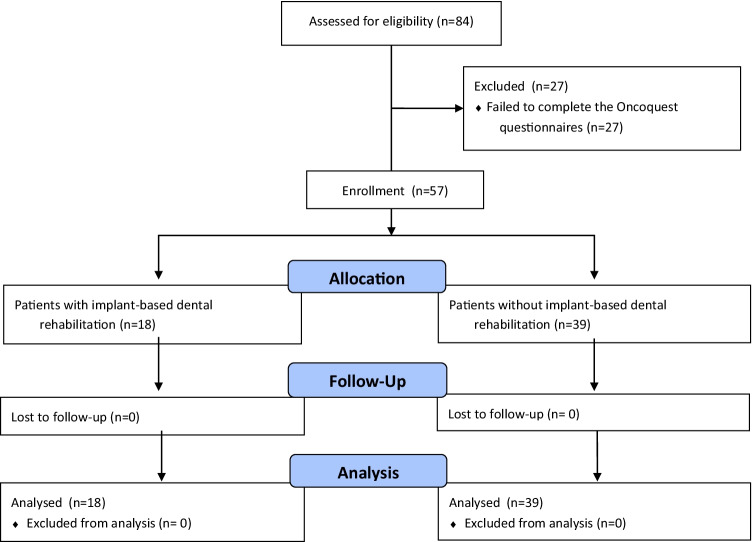
Flow diagram of the included head and neck cancer patients who had undergone maxillofacial reconstruction with a fibula free flap between January 2006 and October 2017

Out of 96 patients who had undergone maxillofacial FFF reconstruction between January 2006 and October 2017, 84 patients had HRQoL data, of which 57 patients had data at both T_0_ and T_1_. These 57 patients were included in this study, of which 18 patients had undergone IDR after FFF reconstruction and 39 did not (Fig. 1).

In Table 1, the demographic and clinical characteristics of the included patients are shown. Significantly more patients in the IDR group (16/18) were edentulous in the reconstructed jaw, compared to the group without IDR (23/39; *p* < 0.01). Significantly more maxillary reconstructions were located in the group with IDR (4/18), compared to the group without IDR (*p* = 0.03).

**Table 1:** Demographic, clinical characteristics and histopathological profile of the included patients at the time of maxillofacial reconstruction with a free vascularized fibula flap (data are percentages unless stated otherwise)

	Without IDR	With IDR	Total	*p* value
Number of patients	39	18	57	
Age (years ± SD)	63.2 ± 9.9	61.9 ± 11.8	62.8 ± 10.4	*0.67*
Gender				
Male	23	11	34	*1.00*
Female	16	7	23	
Tobacco				
Never	10	5	15	*0.40*
Active	21	7	28	
Prior	8	6	14	
Alcohol				
Never	13	6	19	*1.00*
Active	23	10	33	
Prior	3	2	5	
ASA				
I0049	31	13	44	*0.74*
III	8	5	13	
Radiotherapy				
No	3	5	8	*0.18*
Pre-operative	5	6	11	
Post-operative	29	7	36	
Both	2	0	2	
Radiation dose (cGy ± SD)	6381.4 ± 461.1	6162.5 ± 562.9	6325.5 ± 492.2	*0.19*
Reconstructed jaw				
Mandible	39	14	53	** < *0.01***
Maxilla	0	4	4	
Post-operative dental state				
Reconstructed jaw				
Edentulous	23	16	39	***0.03***
(Partial) dentate	16	2	18	
Opposing jaw				
Edentulous	21	11	32	*0.78*
(Partial) dentate	18	7	25	
Number of osteotomy (min–max)	1.5 (0–3)	1.8 (1–3)	1.6 (0–3)	*0.08*
Type of mandibular defect^*^				
Class I	10	1	11	*-*
Class II	11	4	15	
Class III	14	9	23	
Class IV	4	0	4	
Type of maxillary defect †				
IIc		1	1	*-*
IIIb		1	1	
IIId		1	1	
IVc		1	1	
Disease stage				
I/II	6	2	8	*1.00*
III/IV	33	15	48	
Cancer type				
Squamous cell carcinoma	39	17	56	*0.32*
Sarcoma	0	1	1	

*IDR* implant-based dental rehabilitation. Note: For statistical analysis, radiotherapy was dichotomized to radiotherapy and no radiotherapy; tobacco and alcohol use was dichotomized to yes and no. For one patient, the disease stage could not be found

^*^Type of mandibular defect according to Brown et al. (2016)

^†^Type of maxillary defect according to Brown et al. (2010)

Among the 18 patients who received IDR, in total 55 dental implants were placed in the FFFs, with an average of 4.6 dental implants per patient (range: 3–7). Most patients (*n* = 17) received a removable prosthetic construction (bar-retained, *n* = 12; locator-retained, *n* = 4) and achieved a functional dental rehabilitation. One patient received a fixed prosthesis (solitary crowns, *n* = 1). Data regarding the dental implantation procedure and rehabilitation timeline have been previously published [11].

In Table 2, results on the EORTC QLQ-C30 scales are summarized per time assessment. The scales measuring emotional functioning (*p* = 0.01), cognitive functioning (*p* = 0.01) and diarrhoea (*p* = 0.01) were significantly better at T_0_ for patients in the group with IDR, compared to the group without IDR. In Table 3, results on the EORTC QLQ-H&N35 scales are summarized per time assessment. Pain killers were less frequently used at T_0_ for patients in the group with IDR, compared to the group without IDR (*p* = 0.04).

**Table 2:** Within-subject analysis, cross-sectional analysis at T_0_ and comparison of the mean changes in EORTC QLQ-C30 scales for patients who had undergone implant-based dental rehabilitation after FFF reconstruction and those who did not at T_0_ and T_1_

		Without IDR (*n* = 39), mean ± SD	Within-subject, *p* value	With IDR (*n* = 18), mean ± SD	Within-subject, *p* value	Cross-sectional analysis, *p* value	Between-subject, *p* value
Global health status^a^	T_0_	58.0 ± 21.2	0.6	64.3 ± 25.8	0.71	0.42	0.99
	T_1_	61.5 ± 26.8		67.9 ± 25.0			
*Functional scales*^*a*^							
Physical functioning	T_0_	76.0 ± 23.4	0.5	85.7 ± 22.5	0.77	0.25	0.82
	T_1_	71.3 ± 25.6		83.6 ± 12.7			
Role functioning	T_0_	58.0 ± 23.5	0.83	69.0 ± 35.1	0.16	0.4	0.41
	T_1_	60.1 ± 34.8		84.6 ± 15.9			
Emotional functioning	T_0_	55.0 ± 27.6	**0.01**	76.8 ± 21.5	0.71	**0.01**	0.20
	T_1_	73.7 ± 26.1		80.1 ± 24.4			
Cognitive functioning	T_0_	76.7 ± 21.5	0.75	92.9 ± 10.8	0.37	**0.01**	0.41
	T_1_	78.8 ± 24.0		85.9 ± 26.2			
Social functioning	T_0_	72.7 ± 29.2	0.95	75.0 ± 35.5	0.79	0.82	0.79
	T_1_	72.2 ± 29.1		78.2 ± 28.4			
*Symptom scales*^*b*^							
Fatigue	T_0_	42.6 ± 32.2	0.42	28.6 ± 29.5	0.52	0.19	0.97
	T_1_	35.8 ± 30.6		22.2 ± 20.3			
Nausea and vomiting	T_0_	5.3 ± 14.2	0.43	3.6 ± 7.1	0.94	0.67	0.64
	T_1_	8.6 ± 16.2		3.8 ± 10.0			
Pain	T_0_	38.0 ± 25.7	0.30	29.8 ± 35.9	0.32	0.41	0.83
	T_1_	29.3 ± 34.9		17.9 ± 23.0			
Dyspnoea	T_0_	14.7 ± 23.7	0.94	11.9 ± 28.1	0.92	0.76	0.97
	T_1_	15.2 ± 23.7		12.8 ± 16.9			
Insomnia	T_0_	42.7 ± 24.6	**0.03**	26.2 ± 37.4	0.88	0.11	0.19
	T_1_	25.3 ± 33.4		28.2 ± 32.9			
Appetite loss	T_0_	25.0 ± 29.9	0.93	14.3 ± 31.3	0.89	0.3	0.96
	T_1_	24.2 ± 32.6		12.8 ± 21.7			
Constipation	T_0_	6.7 ± 19.2	0.50	7.1 ± 19.3	0.69	0.94	0.95
	T_1_	10.4 ± 21.5		10.3 ± 21.0			
Diarrhoea	T_0_	20.0 ± 30.4	0.32	2.4 ± 8.9	0.16	**0.01**	0.11
	T_1_	12.1 ± 28.6		15.4 ± 32.2			
Financial difficulties	T_0_	10.7 ± 24.9	0.82	11.9 ± 24.8	0.39	0.88	0.44
	T_1_	12.1 ± 23.3		5.1 ± 12.5			

*EORTC* European Organisation for Research and Treatment of Cancer, *FFF* free fibula flap, *SD* standard deviation, *IDR* implant-based dental rehabilitation. Bold printing indicates *p* < 0.05. T_0_ was defined as the period from 6 months before FFF reconstruction until the FFF reconstruction. T_1_ was defined as the period after completing implant-based dental rehabilitation (i.e. after placement of the dental superstructure). For patients who did not undergo implant-based dental rehabilitation, T_1_ was defined as the period after FFF reconstruction

^a^High scores reflect better functioning

^b^High scores reflect more severe symptoms

**Table 3:** Within-subject analysis, cross-sectional analysis at T_0_ and comparison of the mean changes in EORTC QLQ-H&N35 scales for patients who had undergone implant-based dental rehabilitation after FFF reconstruction and those who did not at T_0_ and T_1_

		Without IDR (*n* = 39), mean ± SD	Within-subject, *p* value	With IDR (*n* = 18), mean ± SD	Within-subject, *p* value	Cross-sectional analysis, *p* value	Between-subject, *p* value
*Symptom scales*^a^							
Pain	T_0_	45.2 ± 27.0	0.34	35.7 ± 28.2	0.08	0.30	0.46
	T_1_	37.6 ± 31.5		18.1 ± 18.7			
Swallowing	T_0_	27.1 ± 26.7	0.48	19.0 ± 23.9	0.17	0.38	0.62
	T_1_	33.7 ± 32.9		32.7 ± 26.2			
Senses problems	T_0_	10.1 ± 16.5	0.05	15.5 ± 31.0	0.44	0.50	0.89
	T_1_	22.0 ± 25.2		25.6 ± 35.8			
Speech problems	T_0_	26.8 ± 24.4	0.43	15.9 ± 21.2	0.60	0.18	0.87
	T_1_	20.4 ± 21.6		20.4 ± 21.6			
Trouble with social eating	T_0_	42.9 ± 36.7	0.68	25.0 ± 28.5	0.45	0.15	0.42
	T_1_	38.5 ± 31.1		34.0 ± 32.7			
Trouble with social contact	T_0_	20.9 ± 25.6	0.95	19.0 ± 24.9	0.5	0.83	0.59
	T_1_	21.3 ± 23.9		13.3 ± 15.3			
Less sexuality	T_0_	43.8 ± 37.9	0.95	28.8 ± 33.4	0.31	0.30	0.61
	T_1_	39.5 ± 37.9		16.7 ± 19.7			
*Symptom items* ^a^							
Teeth	T_0_	27.8 ± 32.8	0.29	22.2 ± 32.8	0.89	0.65	0.45
	T_1_	16.7 ± 34.3		24.2 ± 33.6			
Opening mouth	T_0_	41.7 ± 34.3	0.23	31.0 ± 38.0	0.48	0.37	0.91
	T_1_	53.8 ± 38.1		41.0 ± 33.8			
Dry mouth	T_0_	44.0 ± 34.3	0.93	40.5 ± 26.7	0.67	0.74	0.76
	T_1_	44.8 ± 33.5		46.2 ± 39.8			
Sticky saliva	T_0_	43.1 ± 38.7	0.95	31.0 ± 27.6	0.84	0.31	0.92
	T_1_	43.8 ± 40.1		33.3 ± 33.3			
Coughing	T_0_	34.7 ± 32.6	0.47	21.4 ± 28.1	0.16	0.21	0.62
	T_1_	28.1 ± 34.0		7.7 ± 20.0			
Felt ill	T_0_	25.0 ± 34.4	0.81	14.3 ± 25.2	0.86	0.31	0.96
	T_1_	22.9 ± 29.9		12.8 ± 16.9			
Pain killers (%)	T_0_	76.0	**0.04**	42.9	0.12	**0.04**	0.76
	T_1_	50.0		15.4			
Nutritional supplements (%)	T_0_	45.8	0.14	28.6	0.33	0.29	0.89
	T_1_	65.6		46.2			
Feeding tube (%)	T_0_	24.0	0.79	21.4	0.38	0.86	0.31
	T_1_	28.1		7.7			
Weight loss (%)	T_0_	54.2	**0.03**	42.9	**0.01**	0.50	0.67
	T_1_	18.8		7.7			
Weight gain (%)	T_0_	8.3	0.11	14.3	0.46	0.56	0.56
	T_1_	25.0		23.1			

*EORTC* European Organisation for Research and Treatment of Cancer, *FFF* free fibula flap, *SD* standard deviation, *IDR* implant-based dental rehabilitation. Bold printing indicates *p* < 0.05. T_0_ was defined as the period from 6 months before FFF reconstruction until the FFF reconstruction. T_1_ was defined as the period after completing implant-based dental rehabilitation (i.e. after placement of the dental superstructure). For patients who did not undergo implant-based dental rehabilitation T_1_ was defined as the period after FFF reconstruction

^a^High scores reflect more severe symptoms

**Fig. 2:**
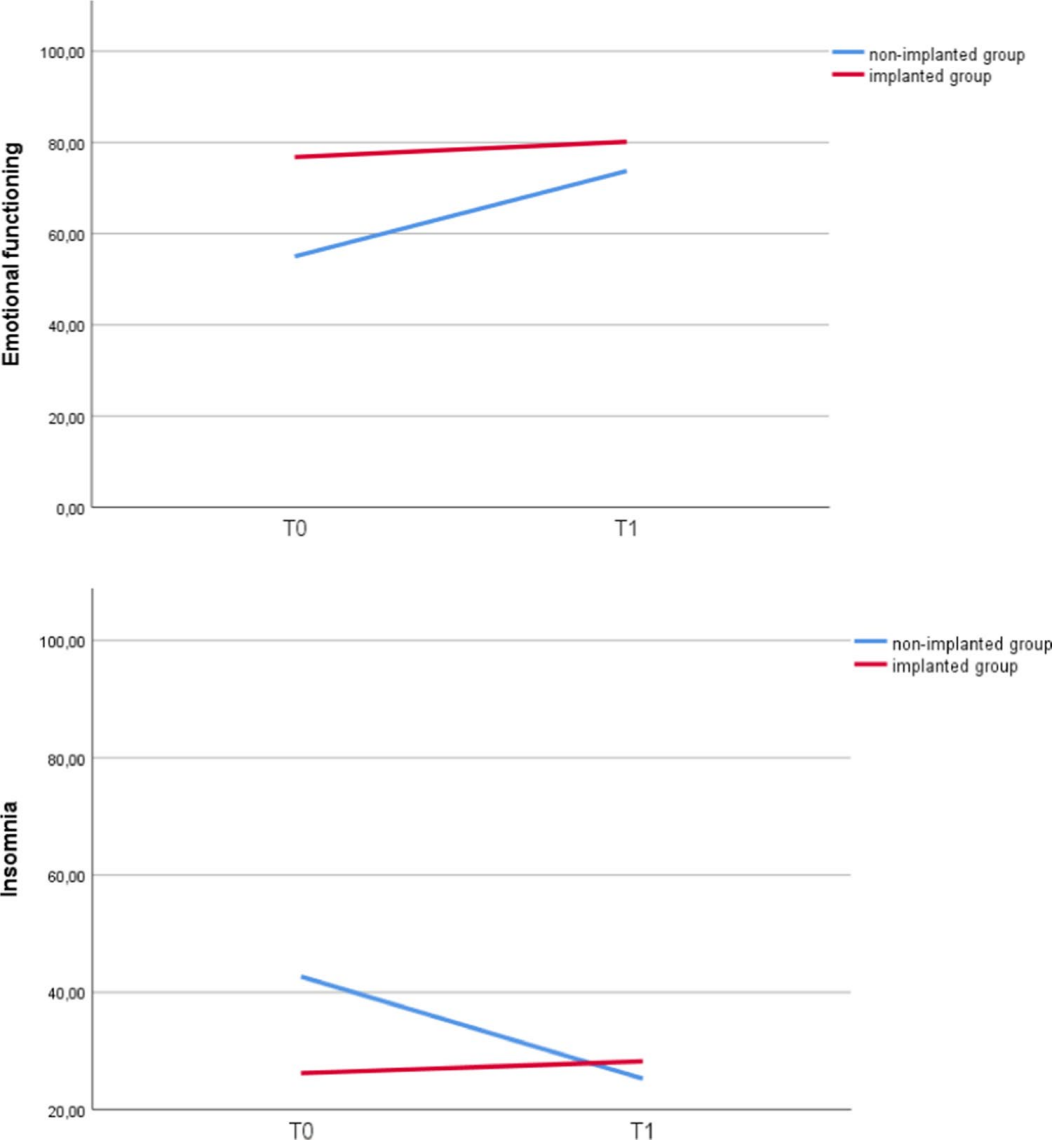
Mean EORTC QLQ-C30 and QLQ-H&N35 scores at T_0_ and T_1_ for scales with statistically significant changes in the within-subject analysis for patients who had undergone implant-based dental rehabilitation after FFF reconstruction and those who did not. EORTC, European Organisation for Research and Treatment of Cancer; FFF, free fibula flap. Patients who had not undergone implant-based dental rehabilitation (blue lines) showed significant differences between T_0_ and T_1_ for the domains emotional functioning (*p* = 0.01) and insomnia (*p* = 0.03). Patients who had undergone implant-based dental rehabilitation (red lines) showed no significant differences between T_0_ and T_1_. T_0_ was defined as the period from 6 months before FFF reconstruction until the FFF reconstruction. T_1_ was defined as the period after completing implant-based dental rehabilitation (i.e. after placement of the dental superstructure). For patients who did not undergo implant-based dental rehabilitation, T_1_ was defined as the period after FFF reconstruction

**Fig. 3:**
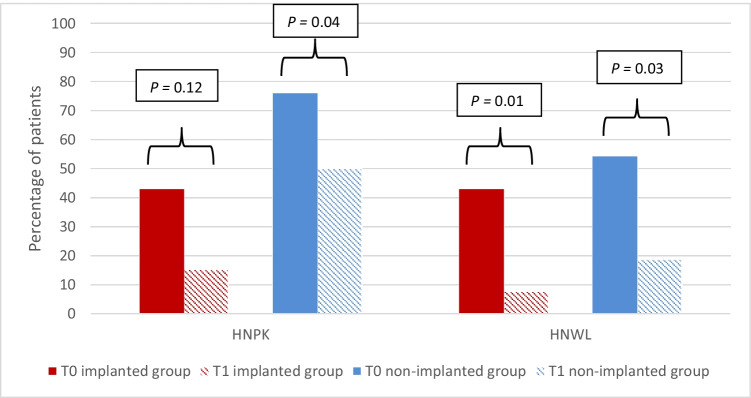
EORTC QLQ-H&N35 scales at T_0_ and T_1_ with statistically significant changes in the within-subject analysis for patients who had undergone implant-based dental rehabilitation after FFF reconstruction and those who did not. EORTC, European Organisation for Research and Treatment of Cancer; FFF, free fibula flap; HNPK, head and neck pain killers; HNWL, head and neck weight loss. Patients who had not undergone implant-based dental rehabilitation (blue) showed significant differences between T_0_ and T_1_ for the domains HNPK (*p* = 0.04) and HNWL (*p* = 0.03). Patients who had undergone implant-based dental rehabilitation (red) showed significant differences between T_0_ and T_1_ for the domain HNWL (*p* = 0.01). T_0_ was defined as the period from 6 months before FFF reconstruction until the FFF reconstruction. T_1_ was defined as the period after completing implant-based dental rehabilitation (i.e. after placement of the dental superstructure). For patients who did not undergo implant-based dental rehabilitation, T_1_ was defined as the period after FFF reconstruction

The results of the within-subject analysis of the EORTC QLQ-C30 and EORTC QLQ-H&N35 scales are shown in Tables 2 and 3. Patients in the group with IDR showed no significant differences between T_0_ and T_1_ for all scales of the EORTC QLQ-C30. In the EORTC QLQ-H&N35, weight loss was significantly less at T_1_ compared to T_0_ (95% confidence interval (CI), 0.06–0.68; *p* = 0.01). Patients in the group without IDR had significant better scores at T_1_ compared to T_0_ for the domains emotional functioning (95% CI, 4.51–32.96; *p* = 0.01), insomnia (95% CI, − 33.3 to − 1.53; *p* = 0.03), pain killers (95% CI, 0.1–0.10; *p* = 0.04) and weight loss (95% CI, 0.04–0.88; *p* = 0.03). The course of these domains (emotional functional, insomnia, pain killers and weight loss) is plotted in Figs. 2 and 3.

There were no significant differences in the mean changes of all the EORTC QLQ-C30 and EORTC QLQ-H&N35 scores between T_0_ and T_1_ for patients who had undergone IDR compared to those who did not (Tables 2 and 3).

## Discussion

To date there is limited evidence on patient-reported outcomes of IDR in terms of HRQoL with validated questionnaires [9]. This study evaluated the course of HRQoL in HNC patients who had undergone IDR after maxillofacial reconstruction with an FFF and compared it to those who did not. In our cross-sectional analysis, patients who had undergone IDR seem to have better HRQoL at T_0_, compared to those who did not. However, only few domains in the EORTC QLQ-C30 and QLQ-H&N35 showed significance. These differences are probably the effect of our selection criteria for patients who commenced IDR. In a study population of 38 patients who had undergone FFF reconstruction of which 23 patients received dental implants, similar findings were reported using cross-sectional analysis [14]. The major drawback for this specific analysis is the one-time measurement.

Dholam et al. used within-subject analysis to evaluate HRQoL with the EORTC QLQ-C30 and QLQ-H&N35 in 12 patients who had undergone dental implantations after FFF reconstruction.^12^ They reported minimal differences in HRQoL after IDR, compared to the situation before FFF reconstruction. The authors explained this finding by the high expectations regarding treatment outcome most patients had, which could not be achieved. We found similar results using within-subject analysis, as only the domain weight loss reached significance after IDR, compared to the situation before FFF reconstruction.

To better answer the question to what extent IDR may have contributed to HRQoL, we compared the differences in the course of HRQoL between patients who commenced IDR and those who did not. Interestingly, HRQoL seems to marginally change in patients who had undergone dental rehabilitation after FFF reconstruction. And, although baseline HRQoL scores may be poorer in patients who do not commence dental implantation after FFF reconstruction, the course of HRQoL seems to be very similar. This finding is reflected in the statistical analysis, as there were no significant differences in HRQoL in patients who commenced IDR, compared to patients who did not. Furthermore, the clinical relevance of these marginal changes in HRQoL scores is debatable.

One prospective clinical trial reported a significant improvement in HRQoL after IDR in patients who had undergone FFF reconstruction [16]. However, it is difficult to translate these findings to oncological patients, because 65% of the patients were reconstructed for benign disease and the majority did not receive radiotherapy. Additionally, all edentulous cases were excluded. In our study population, most patients were edentulous and 85% received radiotherapy.

To date, there are no widely accepted instruments to evaluate the effects of oral rehabilitation on HRQoL [20]. And, although the EORTC QLQ-C30 and EORTC QLQ-H&N35 questionnaires are well validated, these questionnaires could lack sensitivity to identify changes in oral HRQoL. For example, both questionnaires do not address problems related to chewing/eating solid food, choking/gaging and dentures. Additionally, HNC patients have endured different life events compared to a healthy individual and may address other significance to oral function.

Although no significance was found, symptom scales directly related to oral function, including swallowing, speech problems and trouble with social eating, seem to increase over time for patients who commenced IDR and those who did not. As emphasised by other authors, these results may not only be caused by functional deficits, but biopsychosocial aspects could have a profound influence on these findings [21, 22]. Interestingly, patients seem to report a minimal increase in problems with their teeth (symptom scale Teeth) after completion of IDR (T_0_, 22.2; T_1_,24.2). In contrast, patients who did not commence IDR seem to report a decrease in problems with their teeth (T_0_, 27.8; T_1_, 16.7). An explanation for this latter finding may be that patients who did not receive dental implants experienced tumour-related problems that impact HRQoL at baseline and improved after oncological therapy.

Patients included in this study had undergone successful ablative surgery and maxillofacial reconstruction with an FFF. The majority of patients were edentulous in the reconstructed jaw, and all patients who started IDR received a technically well-fabricated dental prosthesis. In our institution, these (edentulous) cases are mainly “bone-driven” reconstructed; i.e. the lower border of the usually atrophied mandible is reconstructed aiming at sufficient facial (chin) projection. To give sufficient support to the soft tissues of the cheek and lower lip, we prefer a removable prosthetic construction which can be optimally designed, both functionally and cosmetically. Moreover, a removable prosthesis may give better access for oral cleaning and may benefit the clinical outcome of dental implants.

In our experience, there seem to be other contributing factors that determine oral function besides adequate reconstruction of the oral anatomy, including remaining sensory and motor functions of the (peri-)oral tissues. Interestingly, a recent study found weak correlations between objective tests of masticatory performance, swallowing and patient-reported outcomes [23] Studies are needed on this topic to evaluate the effect of remaining natural dentition, occluding functional units, defect size and defect location on oral function and HRQoL. To optimise remaining oral function, a multidisciplinary approach can be helpful with a maxillofacial prosthodontist, speech therapist, plastic surgeon, oral and maxillofacial surgeon and ENT specialist [24].

Investigating HRQoL in HNC patients who commence IDR after FFF reconstruction is difficult, as the study design is prone for selection bias. With our concept with delayed implant placement, only those patients who are motivated and have relatively good prognosis commenced IDR [10, 11]. This selection bias is illustrated by the significant difference in edentulism in the reconstructed jaw between patients who received dental implants and those who did not. Although, the effect of remaining occluding teeth has not been investigated in patients reconstructed with an FFF, there is evidence that remaining occluding teeth may have a positive effect on masticatory performance in HNC patients [25]. Additionally, it seems that radiotherapy and ASA class III are represented more in the group without IDR. As demonstrated, both factors have a significant impact on HRQoL [16, 26, 27].

We retrospectively analysed HRQoL data, resulting in some heterogeneity in the timepoints of HRQoL collection. Furthermore, the small sample size could have influenced the results of this study and made comparison between and within groups difficult. Future prospective studies should not only focus on more robust data but should also assess HRQoL at predetermined timepoints for patients who undergo dental rehabilitation after FFF reconstruction, particularly, information on HRQoL after finishing the oncologic treatment, compared to HRQoL after completion of IDR.

## Conclusion

Although there were differences in HRQoL before oncological therapy between HNC patients who had undergone IDR after maxillofacial FFF reconstruction and those who did not, there seem to be no significant differences in the course of HRQoL between both groups. Prospective studies on HRQoL with validated, specific questionnaires focusing on oral functioning are necessary on this topic to improve and shape treatment strategies for this specific patient group. Patients should be informed to have realistic expectations regarding the outcome of IDR.

## Data Availability

The authors declare that all data and materials support the published claims and comply with field standards.
